# Rescue and in vitro characterization of a divergent TBEV-Eu strain from the Netherlands

**DOI:** 10.1038/s41598-023-29075-0

**Published:** 2023-02-18

**Authors:** Tabitha E. Hoornweg, Gert-Jan Godeke, Marieke N. Hoogerwerf, Puck B. van Kasteren, Ankje de Vries, Hein Sprong, Georges M. G. M. Verjans, Debby van Riel, Johan H. J. Reimerink, Barry Rockx, Chantal B. E. M. Reusken

**Affiliations:** 1 Center for Infectious Disease Control, National Institute for Public Health and the Environment, Antonie Van Leeuwenhoeklaan 9, 3720 BA Bilthoven, The Netherlands; 2 Department of Viroscience, Erasmus University Medical Center, Dr. Molewaterplein 50, 3015 GE Rotterdam, The Netherlands; 3 Present Address: Department of Biomolecular Health Sciences, Division of Infectious Diseases and Immunology, Faculty of Veterinary Medicine, Utrecht University, Utrecht, The Netherlands

**Keywords:** Pathogens, Virology

## Abstract

Tick-borne encephalitis virus (TBEV) may cause tick-borne encephalitis (TBE), a potential life-threatening infection of the central nervous system in humans. Phylogenetically, TBEVs can be subdivided into three main subtypes, which differ in endemic region and pathogenic potential. In 2016, TBEV was first detected in the Netherlands. One of two detected strains, referred to as Salland, belonged to the TBEV-Eu subtype, yet diverged ≥ 2% on amino acid level from other members of this subtype. Here, we report the successful rescue of this strain using infectious subgenomic amplicons and its subsequent in vitro characterization by comparison to two well-characterized TBEV-Eu strains; Neudoerfl and Hypr. In the human alveolar epithelial cell line A549, growth kinetics of Salland were comparable to the high pathogenicity TBEV-Eu strain Hypr, and both strains grew considerably faster than the mildly pathogenic strain Neudoerfl. In the human neuroblastoma cell line SK-N-SH, Salland replicated faster and to higher infectious titers than both reference strains. All three TBEV strains infected primary human monocyte-derived dendritic cells to a similar extent and interacted with the type I interferon system in a similar manner. The current study serves as the first in vitro characterization of the novel, divergent TBEV-Eu strain Salland.

## Introduction

Tick-borne encephalitis virus (TBEV), a member of genus *Flavivirus*, family *Flaviviridae,* is the causative agent of tick-borne encephalitis (TBE), a potential life-threatening disease in humans. The virus is endemic over a wide area of the Eurasian continent and causes over 10,000 human cases annually^1,2^. Infections with TBEV are often asymptomatic^1–4^, but may lead to a wide variety of symptoms in humans. After initial infection, TBEV spreads systemically and may induce non-specific symptoms including fever, fatigue, headache and myalgia. In some patients the disease resolves after this first phase (monophasic TBE), while in others the virus invades the CNS and causes a second phase of illness characterized by neurological manifestations such as meningitis, paralysis and encephalitis (biphasic TBE), which may become fatal^1,2^.

Phylogenetically, TBEV can be divided into three main subtypes, which are associated with varying disease severities in humans^1,5,6^. The European subtype (TBEV-Eu) is detected in Europe, Siberia and Korea^4^, and causes a relatively mild form of TBE with case fatality rates below 1%^2,7–9^. The Far Eastern subtype (TBEV-FE) is primarily found in Russia, Northern China and Japan^4^, but has also been detected in several Eastern European countries^5,10–12^. TBEV-FE causes the most severe neurological manifestations and has also been implicated with a hemorrhagic form of TBE^2,13^. For TBEV-FE, case fatality rates of up to 20% have been reported^2,14^. Lastly, the Siberian subtype (TBEV-Sib) is mainly distributed throughout Russia^4^, but has also been detected in Northern and Eastern Europe^15–17^. TBEV-Sib is associated with TBE of intermediate severity, with case fatality rates of 6–8%^14^. Unlike TBEV-Eu, TBEV-Sib and (albeit to a lesser extent) TBEV-FE may cause a chronic form of TBE in humans^1,2^. In addition to the three main subtypes, two novel genetic TBEV variants, the Baikalian (TBEV-Bkl) and Himalayan (TBEV-Him), were recently proposed as separate subtypes based on their distant phylogenetic relationship with known TBEV subtypes^18–20^, differing at least 11% (TBEV-Bkl) and 15% (TBEV-Him) at nucleotide level from other TBEV subtypes. For both proposed subtypes, associated disease severity and mortality rates are still unknown.

In 2015, first evidence of TBEV circulation in the Netherlands was found^21^, followed by recognition of the first autochthonous TBE cases in 2016^22–26^. In the same period, two distinct TBEV-Eu strains were detected in ticks collected at two separate locations in the Netherlands^21,22^. One of the strains, NL/UH 2016 (Genbank accession number MH021184) was detected in a tick retrieved from a TBE patient that hiked at National Park ‘Utrechtse Heuvelrug’ in the center of the country. This strain was highly similar to TBEV-Eu strains already known and phylogenetically clustered with strains detected in Sweden, Finland and Slovenia. The second strain, NL (Genbank accession number LC171402), was detected in ticks caught in 2015 at National Park ‘Sallandse Heuvelrug’ in the east of the Netherlands. This strain—hereafter referred to as ‘Salland’ to discern it from the other TBEV-Eu strain detected in the Netherlands—was most closely related to strains of the TBEV-Eu subtype, yet diverged from all known TBEV strains by at least 8.5% at nucleotide level and 2% at the amino acid level. Late 2019, a second TBEV-Eu strain highly similar to the divergent TBEV-Eu strain ‘Salland’ was detected in the United Kingdom^27^.

Severity of TBE depends on factors including host age, genetic predisposition and immune status, amount of virus inoculated and the TBEV strain causing infection^2^. Among all TBEV subtypes highly and non-pathogenic strains have been described, however the molecular basis for the observed differences in pathogenicity is still poorly understood^28–30^. As the novel TBEV-Eu strain Salland was found to be divergent from all other known TBEV-Eu strains, we aimed to isolate this virus in vitro to assess its pathogenic potential. Here, we describe the successful rescue of TBEV Salland by use of Infectious Subgenomic Amplicons (ISA) and its subsequent in vitro comparison with two TBEV-Eu strains of well-known pathogenicity, Neudoerfl (TBEV-Eu strain of low to medium pathogenicity) and Hypr (highly pathogenic TBEV-Eu strain). Our data suggests that the novel TBEV-Eu strain Salland may be a TBEV strain with pathogenic potential.

## Results

### Genetic distance of TBEV Salland to other TBEV-Eu strains

**Figure 1:**
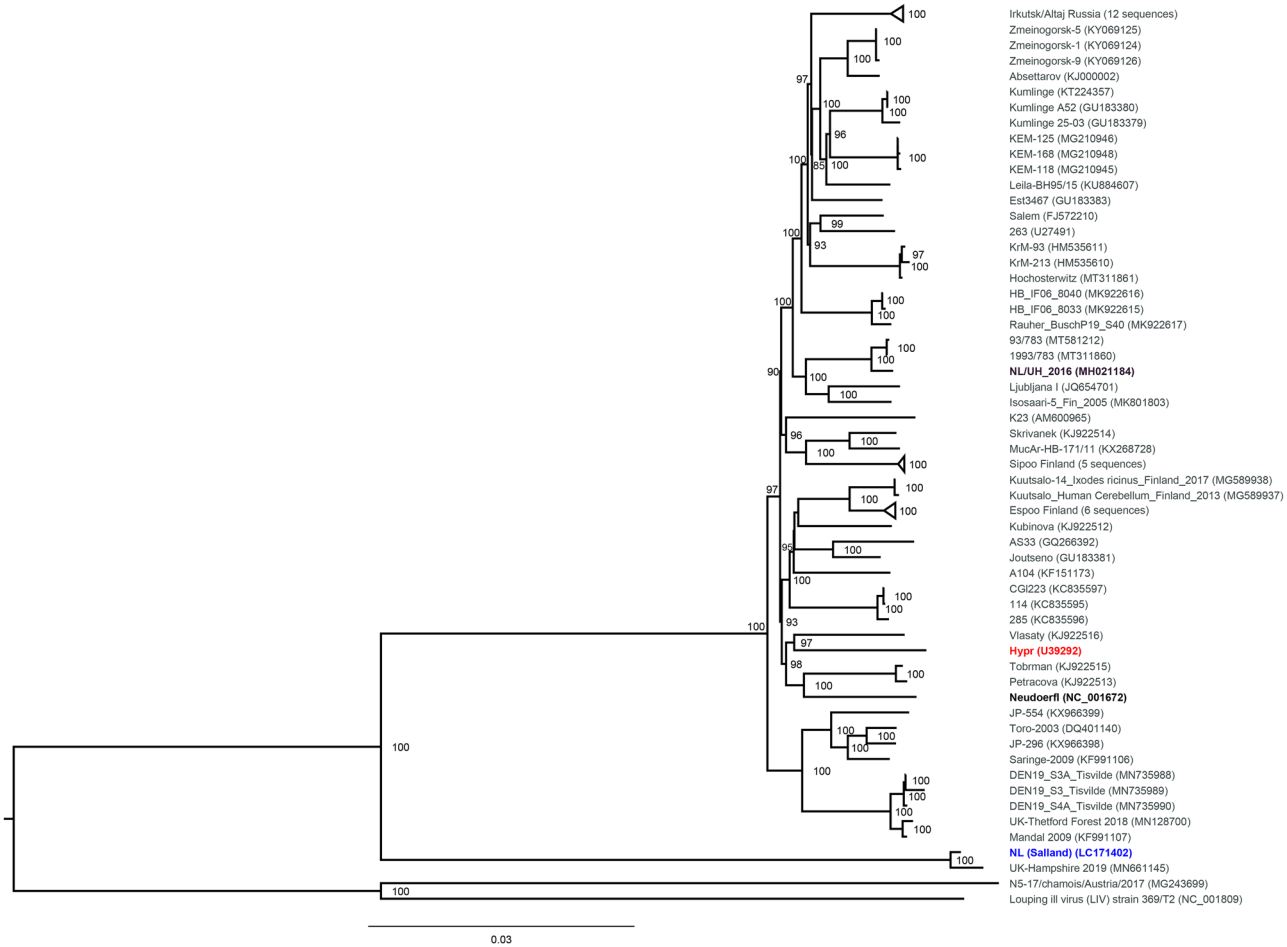
Maximum Likelihood Tree showing the phylogenetic relationships within the TBEV-Eu subtype. Complete genome open reading frame sequences were retrieved from GenBank and aligned in Jalview using the Mafft algorithm. The tree was constructed with IQTree using the GTR + F + R3 evolutionary model and 1000 bootstrap replicates. The resulting tree was visualized and edited in FigTree. The tree was rooted using Louping Ill Virus and N5-17/chamois/Austria/2017, a strain that was annotated as a TBEV sequence in GenBank but was found to be more closely related to LIV, as the outgroup. All bootstrap values ≥ 70 are shown. The two TBEV sequences detected in the Netherlands plus the two reference strains used in this study are highlighted; Salland in blue, NL/UH in purple, Neudoerfl in black and Hypr in red.

In order to visualize the mutual relatedness of TBEV sequences that were detected in TBEV-positive ticks in the Netherlands and determine their phylogenetic relationship with known TBEV-Eu strains, a maximum likelihood tree including all full length TBEV-Eu strains currently deposited in GenBank was inferred (Fig. 1). As expected, the TBEV-Eu strain detected in ticks from National Park ‘Utrechtse Heuvelrug’ in 2016 (NL/UH_2016) was positioned within the large cluster of TBEV-Eu sequences, being most related to a sequence detected in Sweden in 1993. Notably, the TBEV-Eu strain 'Salland’ detected in 2015 was highly related the TBEV-Eu strain detected in Hampshire in 2019, yet both strains diverged from all other TBEV-Eu strains. The strain N5-17/chamois/Austria/2017, annotated as a TBEV strain in GenBank, clustered with Louping Ill Virus (LIV), a related tick-borne flavivirus that was included as the outgroup, rather than with the TBEV-Eu sequences. A BLAST search on this sequence showed that this strain was more related to Spanish Goat Encephalitis Virus (SGEV; 94.9% identity) and LIV (92.1% identity) than to the currently known TBEV-Eu strains (87.0–87.6% identity). This was in contrast to the Salland and Hampshire strains, which showed higher levels of nucleotide identity to the large cluster of TBEV-Eu strains (90.9–91.5%) than to related tick-borne flaviviruses such as SGEV and LIV (≤ 87.7% identity).

The data infer that the novel TBEV-Eu strains from Salland and Hampshire differ 8.5–9.1% from all other TBEV-Eu sequences at nucleotide level, translating into an amino acid (AA) difference of 2.0 to 3.4% in the viral polyprotein. To characterize whether these AA changes are randomly distributed or primarily clustered within specific viral proteins, the translated polyprotein sequence of the TBEV-Eu strain Salland was compared to the TBEV-Eu prototype strain Neudoerfl (Fig. S1). Overall, 82 AA substitutions, but no inserts or deletions, were found in the complete polyprotein of 3414 AAs, corresponding to a genetic distance of 2.4%. Assessment of the individual viral proteins showed the percentage AA substitutions ranged from 0.8% (NS4A) to 3.9% (NS2A). Both percentages fell within two standard deviations (SD = 1.41%) from the mean genetic distance (2.4%), suggesting there was no significant clustering of amino acid substitutions in specific viral proteins.

### Rescue of TBEV Salland strain using infectious subgenomic amplicons

To characterize the divergent TBEV-Eu strain Salland at the phenotype level, we initially aimed to isolate the virus by cell culture. For this purpose, in 2016, a total of 2365 ticks were caught at the same location in National Park ‘Sallandse Heuvelrug’ where TBEV-infected ticks were detected in 2015. Ticks were divided in 275 pools consisting of either nymphs (2190 in 223 pools), females (78 in 29 pools) or males (97 in 23 pools) and tested for TBEV by real-time quantitative reverse transcription PCR (qRT-PCR). None of the pools tested positive for TBEV, preventing isolation of infectious TBEV.

Consequently, we aimed to rescue the TBEV-Eu strain Salland using reverse genetic techniques, for which the complete viral genome sequence is required. Compared to available full-length TBEV genomes, the sequence of the TBEV-Eu strain Salland deposited in GenBank (LC171402) lacked 21 and 95 nucleotides (nt) at the 5′ and 3′-end of the genome, respectively. To resolve these sequences, amplicons previously used for Sanger sequencing of the (near-complete) genome were subjected to Illumina MiSeq sequencing. Using this procedure, 14 and 93 additional nucleotides of the 5′- respectively 3′-UTR could be resolved (GenBank accession number ON502378), leaving only 7 and 2 nucleotides at the extreme ends of the genome unsolved. As the extreme ends of the TBEV genome are highly conserved^31^, both within and between TBEV subtypes (Tables S1 and S2), the genome was completed by adding 5′-AGATTTT-3′ (5′-UTR; conserved in 97% of tick-borne flavivirus genomes) and 5′-CT-3′ (3′-UTR; conserved in 100% of tick-borne flavivirus genomes) to the extreme ends of the genome. The resultant ‘complete’ genome is deposited at GenBank (GenBank accession number BK061374).

Infectious subgenomic amplicons^32^ of the complete genome of the TBEV-Eu strains Salland and Hypr, the latter taken along as a positive control, were designed as described in “Materials and methods” and transfected into baby hamster kidney fibroblast BHK-21 cells. Five to six days post-transfection, cytopathic effect (CPE; visible as an increased number of rounded or detached cells compared to the mock transfected cells) was clearly visible in TBEV amplicon-transfected cells. High titers of infectious recombinant virus (6 × 10^8^ and 6 × 10^7^ plaque forming units (PFU)/ml for Salland and Hypr, respectively) were detected in the supernatant as determined by plaque assay. Successful recombination of the viral genomes was confirmed by PCR using primer combinations that spanned the recombination sites, and identity of the strains (without acquisition of additional mutations) was confirmed by Illumina MiSeq Sequencing (data not shown).

### TBEV Salland strain produces as much infectious progeny as Hypr in A549 cells, but induces less cytopathic effect

We next aimed to compare the phenotypic characteristics of the rescued TBEV-Eu strain Salland to two well-characterized TBEV-Eu strains of known pathogenicity. For this, the TBEV-Eu reference strain Neudoerfl, which was isolated from ticks in 1971 and shows low to medium pathogenic potential^33^, was acquired through the European Virus Archive, whereas Hypr, a TBEV-Eu strain originally isolated from the blood of a deceased child in 1953 and which is considered highly virulent^29,33–35^, was acquired using Infectious Subgenomic Amplicons.

**Figure 2:**
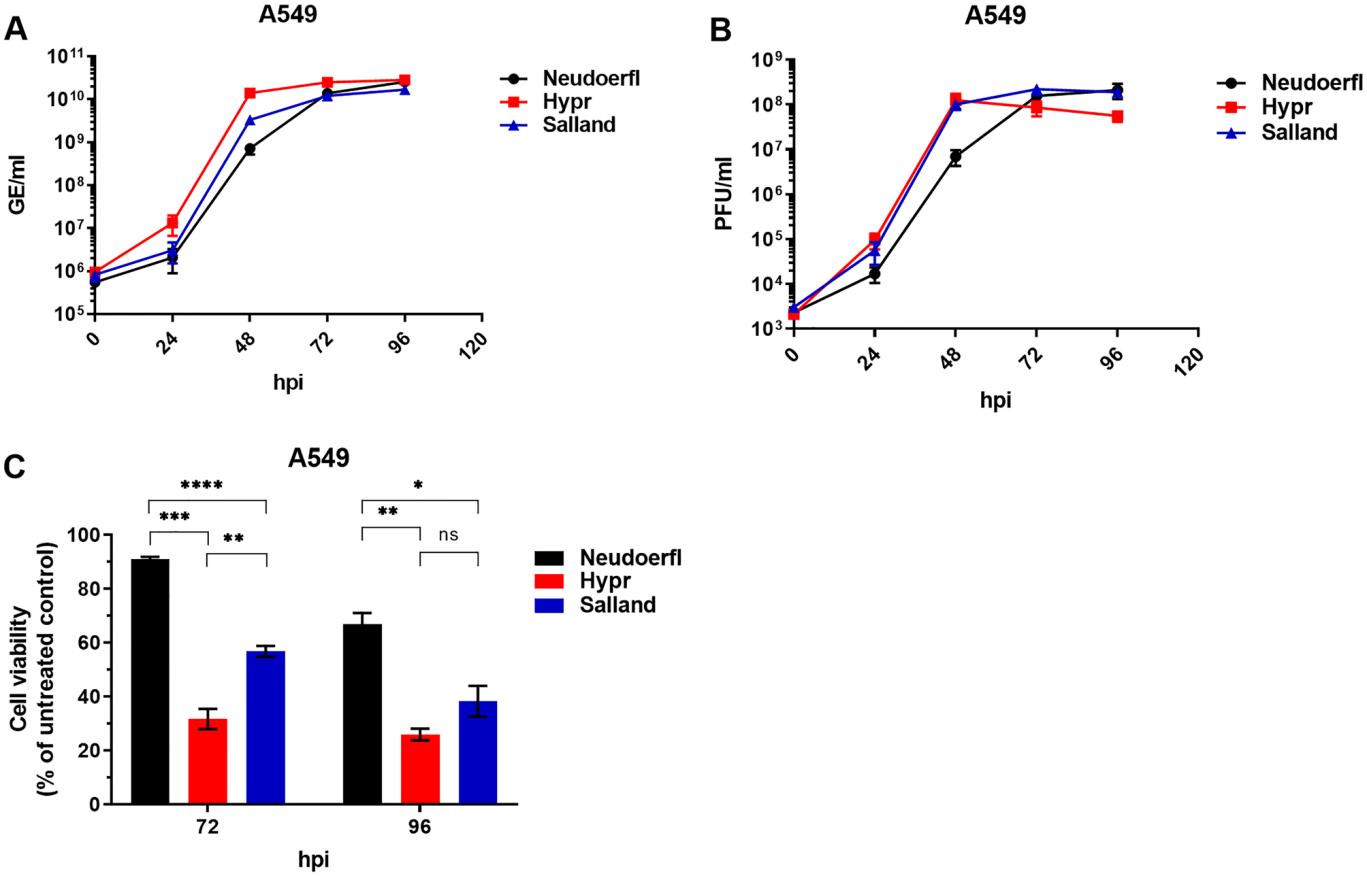
Viral growth kinetics and CPE-induction on A549 cells. A549 cultures were infected with TBEV-Eu strains Neudoerfl, Hypr or Salland at MOI 0.01 and sampled at indicated timepoints post infection. All values indicate mean ± SEM. (**A**) GE/ml production as assessed by qRT-PCR. Experiments were performed three times in triplicate. (**B**) PFU/ml as assessed by plaque assay. Experiments were repeated three times. (**C**) Cell viability of TBEV infected cultures as determined by PrestoBlue assay. Experiments were performed three times in eightfold and normalized to non-infected control cells. Statistical analysis was performed using an unpaired students t-test in GraphPad Prism; *(p ≤ 0.05), **(p ≤ 0.01) ***(p ≤ 0.001), ****(p ≤ 0.0001), ns = non-significant.

Initial growth curves were performed on human alveolar epithelial A549 cells, which are highly permissive to flavivirus infection, including TBEV^29,36^, and retain innate immune function^37–40^. Figure 2A shows the viral growth kinetics over a period of 96 h as based on genome equivalents quantified by qRT-PCR. While all three TBEV strains reach similar peak titers (~ 10^10^ GE/ml), Hypr reached peak titers considerably faster than Neudoerfl (48 h post infection (hpi) versus 72–96 hpi, respectively), while Salland showed intermediate growth kinetics, reaching peak titers at 72 hpi. Upon quantification of infectious titers by plaque assay, growth curves of Hypr and Salland largely overlapped, with both strains producing infectious particles considerably faster than Neudoerfl (Fig. 2B). While all three strains eventually reached comparable peak titers (~ 10^8^ PFU/ml), peak titers were reached at 48 hpi for Hypr and Salland and 72–96 hpi for Neudoerfl.

While performing the TBEV growth curves in A549, it was noted that Hypr and Salland induced CPE at earlier time points post infection than Neudoerfl (Fig. S2), which may be in line with their faster replication kinetics. To compare TBEV-induced CPE, cell viability was quantified at 72 and 96 hpi (Fig. 2C). While 91% of the cells in Neudoerfl-infected wells were still viable at 72 hpi, cell viability had dropped to 32% and 57% within the Hypr and Salland-infected wells, respectively. At 96 hpi, a clear decrease in cell viability was also observed within the Neudoerfl-infected wells (67%), yet cell viability measured in the wells infected with Hypr or Salland was again significantly lower (26% and 38%, respectively).

### TBEV Salland shows comparable growth kinetics and cytopathic effect to the highly pathogenic TBEV-Eu strain Hypr in human brain resident cell lines

**Figure 3:**
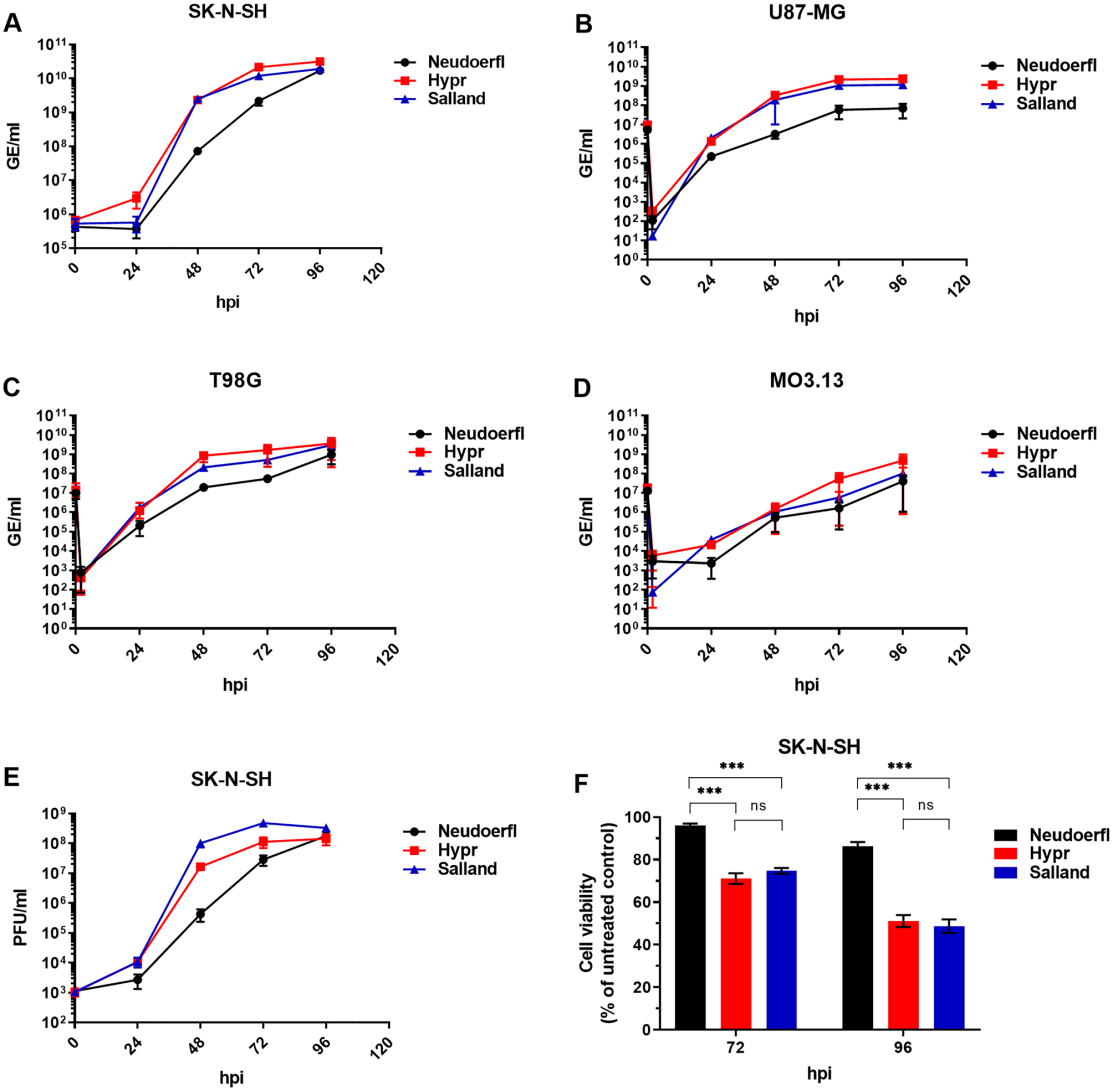
Viral growth kinetics and CPE-induction on neuronal cell lines. SK-N-SH, U87-MG, T98G and MO3.13 cultures were infected with the different TBEV-Eu strains at MOI 0.01 and sampled at indicated timepoints post infection. All values indicate mean ± SEM. GE/ml production on (**A**) SK-N-SH, (**B**) U87-MG, (**C**) T98G and (**D**) MO3.13 as assessed by qRT-PCR. Experiments were performed (**A**) three times in triplicate or (**B**–**D**) two times in triplicate. (**E**) PFU/ml production on SK-N-SH cells as assessed by plaque assay. Experiments were repeated three times. (**C**) Cell viability of TBEV infected cultures as determined by PrestoBlue assay. Experiments were performed three times in eightfold and normalized to non-infected control cells. Statistical analysis was performed using unpaired students t-test in GraphPad Prism; ***(p ≤ 0.001), ns = non-significant.

As TBEV is a neurotropic virus, we next compared growth kinetics of the different TBEV-Eu strains in human brain resident cells. For this, human cell lines SK-N-SH (neuroblastoma), U87-MG (glioblastoma), T98G (multiforme glioblastoma), MO3.13 (oligodendrocytoma) were infected at an multiplicity of infection (MOI) of 0.01 and viral titers (GE/ml) were assessed over time by qRT-PCR (Fig. 3A–D). In the neuro- and glioblastoma cell lines (Fig. 3A–C), both the virulent TBEV-Eu strain Hypr and novel TBEV-Eu strain Salland showed comparable growth kinetics, while the mildly pathogenic Neudoerfl replicated slower (Fig. 3A–C) and, in U87-MG, to lower titers (Fig. 3B). In the oligodendrocytoma cell line MO3.13, replication of all three virus strains appeared inefficient. Although Hypr was observed to grow to the highest titers in this cell line, observed differences were small.

As all three TBEV strains replicated most efficiently in the neuroblastoma cell line SK-N-SH, this cell line was used for further characterization. Figure 3E shows the amount of infectious virus produced for the three virus strains as quantified by plaque assay. While comparable amounts of genome equivalents were detected for both Hypr and Salland in SK-N-SH cells, consistently higher infectious virus titers were measured for Salland in these cells, with the most prominent difference (sixfold) observed at 48 hpi. In line with the GE/ml titers shown in Fig. 3A, Neudoerfl grew slower than both other virus strains, with ~ 40 and ~ 240-fold lower infectious titers at 48 hpi compared to Hypr and Salland, respectively, but eventually reached comparable peak titers to the other viruses (~ 10^8^ PFU/ml) at 96 hpi.

While for Hypr and Salland CPE was visible from 72 hpi, almost no CPE was observed for Neudoerfl until 96 hpi (Fig. S3). In line with these observations, 96% cell viability was detected within the Neudoerfl-infected wells at 72 hpi, while a significantly lower percentage of cells remained viable upon infection with Hypr or Salland (71% respectively 75%; Fig. 3F). At 96 hpi, cell viability decreased to 86% in Neudoerfl-infected wells, while only ~ 50% cell viability was detected within the wells infected with Hypr (51%) or Salland (49%).

### Human moDCs are efficiently infected by all three TBEV-Eu strains

**Figure 4:**
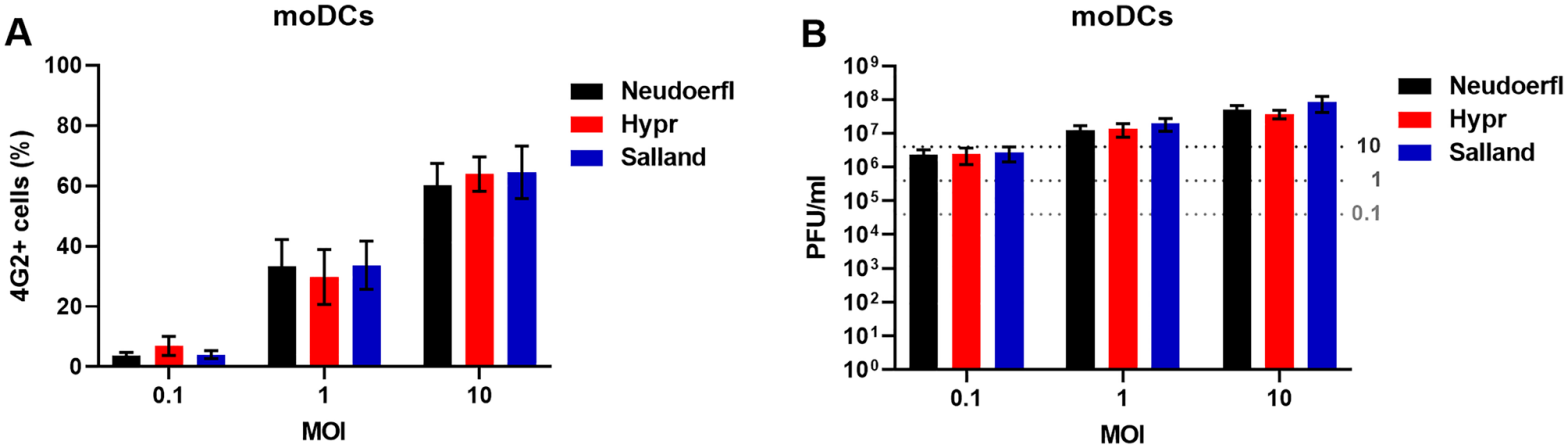
TBEV infection of moDCs. moDCs of three individual donors were infected with the different TBEV strains at various MOIs for 26 h. (**A**) Percentage of TBEV infected cells as quantified by fluorescent microscopy. (**B**) Infectious virus production as assessed by plaque assay. The inoculum titers per infection condition (MOI) are indicated by dashed grey lines. All values indicate average of three donors ± SEM.

As dendritic cells of the skin are assumed to be one of the first cell types to get infected after a bite of a TBEV-infected tick, we next assessed whether all three TBEV-Eu strains can efficiently initiate infection in this cell type. For this, monocyte-derived DCs (moDCs) were generated from three healthy individual donors and subsequently infected at low (0.1), intermediate (1) and high (10) MOIs for one replication cycle (26 h). Figure 4A shows the number of infected cells quantified by microscopy, while Fig. 4B shows produced infectious viral titers. All TBEV strains were observed to infect moDCs efficiently, with no significant difference in number of infected cells or produced viral progeny observed between the different viral strains.

### The type I interferon response is triggered to a comparable extent by all three TBEV strains

**Figure 5:**
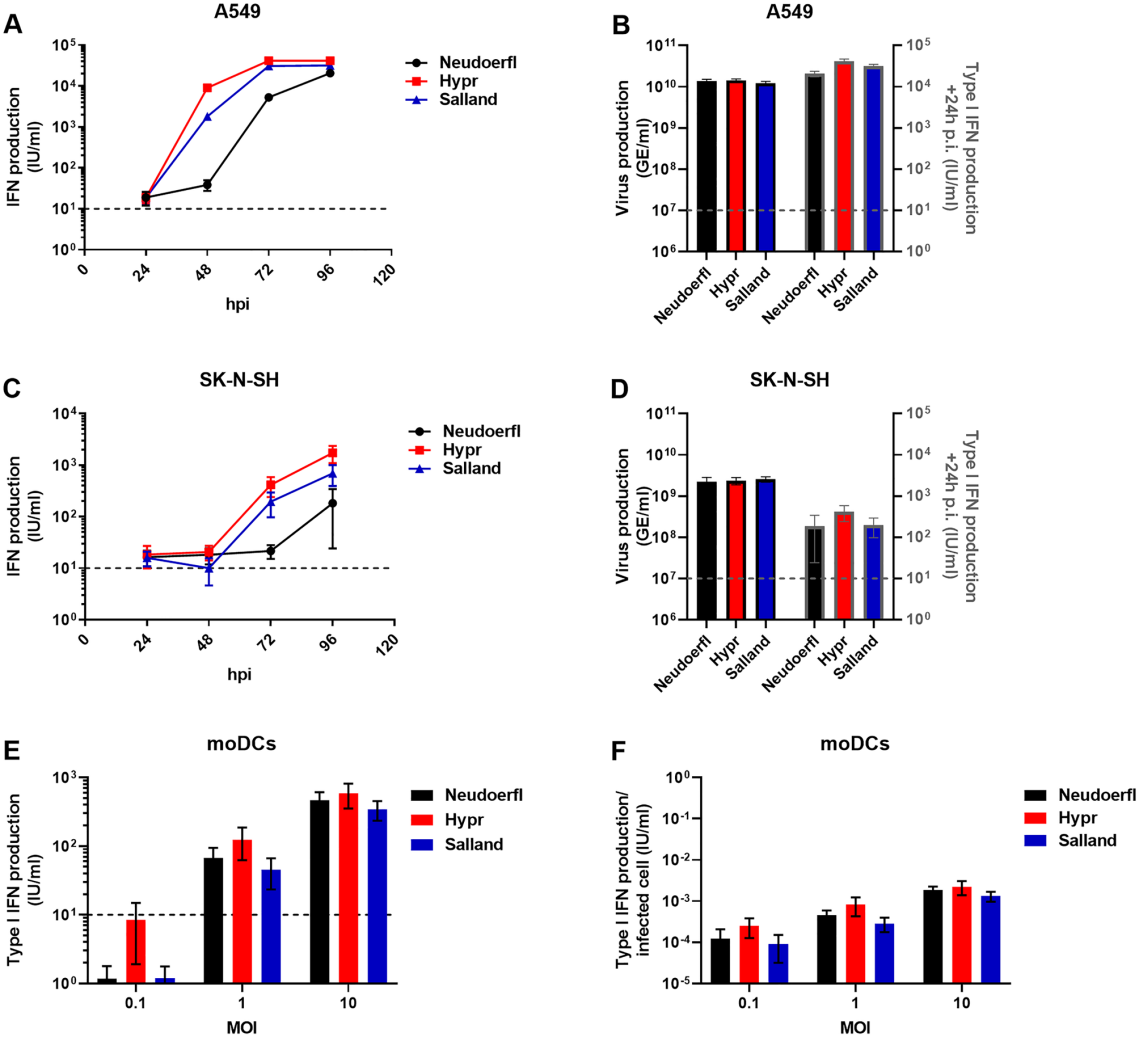
IFN-α/β production upon TBEV infection of A549, SK-N-SH, and moDCs. IFN-α/β production in supernatants from viral growth curves (Figs. 2A,B and 3A,E) and moDC infection experiments (Fig. 4) was measured using HEK-Blue™ IFN-α/β reporter cells. (**A**,**C**) IFN-α/β production in response to TBEV replication in A549 (**A**) or SK-N-SH (**C**) cells. IFN production was assessed in duplicate for two replicates from three individual experiments. (**B**,**D**) Comparison of GE/ml production (bars lined in black and left Y-axis) with IFN-α/β production (bars lined in grey and right Y-axis) with a 24 h lag time for (**B**) A549 and (**D**) SK-N-SH cells. (**E**) IFN-α/β production in supernatants harvested 26 hpi from TBEV-infected moDCs. (**F**) IFN-α/β production per infected moDC. Number of infected cells were calculated based on the percentage of infected cells determined by microscopy (Fig. 4A) and the total number of cells per well. All values depict mean ± SEM. Dashed lines indicate the limit of IFN detection in the experimental setup.

The type I interferon (IFN-I) system is known to be important to control TBEV replication both in vivo and in vitro^41–43^. To test whether the IFN-I response is efficiently triggered upon infection with the different TBEV strains, we quantified the amount of IFN-α/β secreted into the supernatants during viral replication. In A549 cells, clear differences in IFN-α/β production were observed at 48 and 72 hpi between Hypr and Salland versus Neudoerfl (Fig. 5A). As observed differences may be due to strain-specific differences in replication kinetics, CPE, or induction of the IFN-α/β response, the data was normalized for viral replication (GE/ml) while taking into account that TBEV is able to delay the IFN-α/β response for approximately 24 h after infection^29,44^. Approximately 24 h after similar numbers of genome copies were produced (~ 10^10^ GE/ml, detected 48 hpi for Hypr and 72 hpi for Salland and Neudoerfl), comparable IFN-α/β titers were observed (Fig. 5B).

Notable differences in IFN- α/β production were also observed after infection of SK-N-SH cells with the different TBEV strains (Fig. 5C). Yet, similar to the IFN- α/β responses produced by A549 cells, 24 h after comparable numbers of genome copies were produced (~ 2 × 10^9^ GE/ml, detected at 48 hpi for Hypr and Salland and 72 hpi for Neudoerfl), IFN-α/β production only differed slightly (2.4-fold, Fig. 5D).

Finally, the IFN-α/β response was measured upon infection of moDCs. Figure 5E shows the amount of IFN-α/β produced 26 hpi, while Fig. 5F shows the IFN-α/β production normalized to the number of infected cells. Normalization to genome equivalents per ml as done in Fig. 5B,C was not possible as viral progeny titers could not be determined due to limited sample volume available. While IFN-α/β production clearly increased upon infection with higher MOIs, no significant differences in IFN-α/β production were observed between the different TBEV strains. Together, the data presented above suggests that all three TBEV strains trigger the IFN-I response to a similar extent.

### All three TBEV strains interfere with the IFN-I signaling cascade with similar efficiency

**Figure 6:**
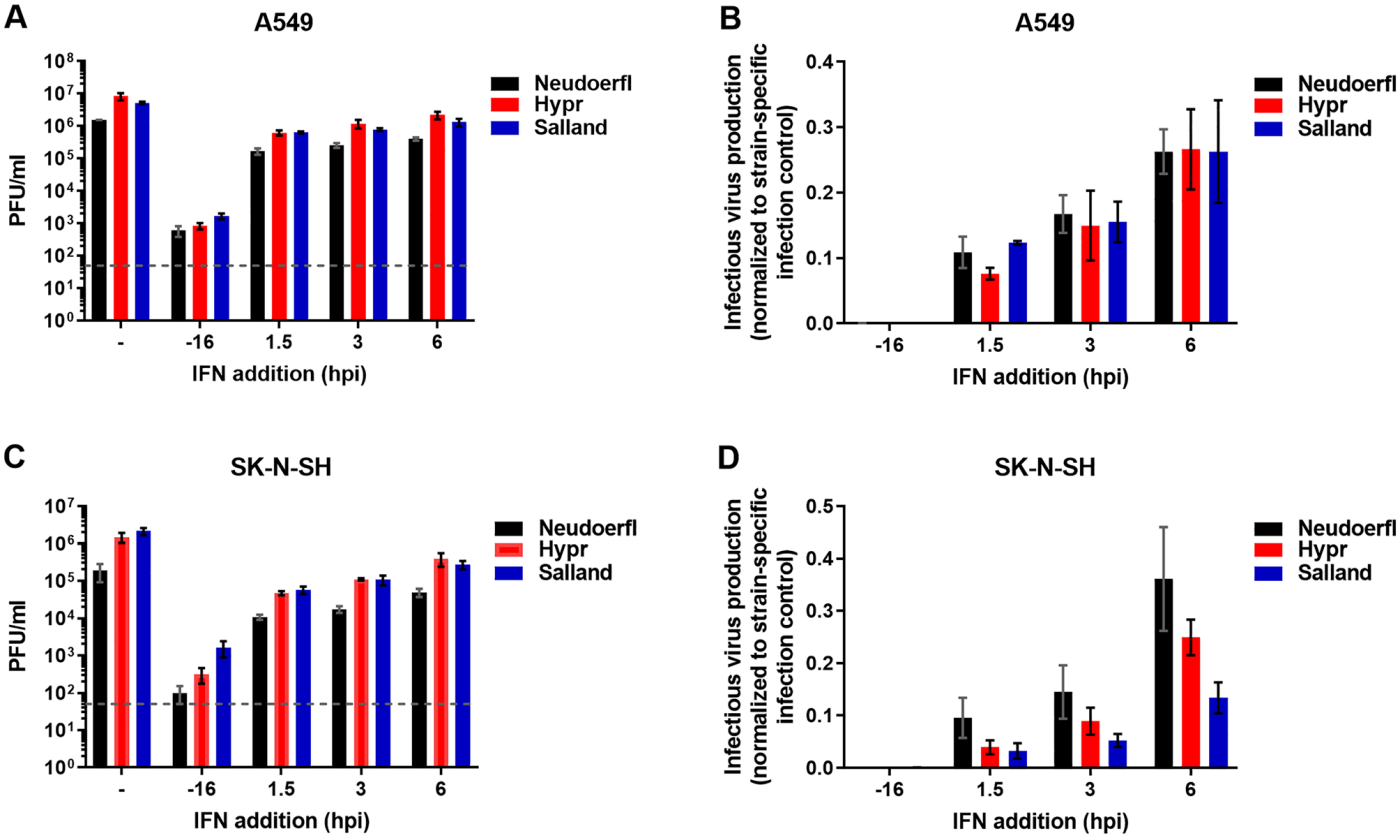
Sensitivity of the different TBEV strains to exogenous IFN-α. A549 (**A**,**B**) and SK-N-SH (**C**,**D**) cells were infected with MOI 1 for 26 h in the presence or absence of exogenous IFN-α 2a, which was added at different time points pre- and post-infection. (**A**,**C**) Depict infectious virus progeny in harvested supernatants as determined by plaque assay. Dashed lines indicate the limit of detection in the experimental setup. In (**B**,**D**) obtained PFU/ml titers are normalized to infectious titers obtained for the non-treated control. Mean values ± SEM of three individual experiments are shown.

For other neurotropic flaviviruses, increased interference with IFN-I signaling, thereby preventing induction of an antiviral state, has been associated with pathogenicity^45,46^. To check whether the TBEV strains differed in their potential to interfere with IFN-I cascade, A549 and SK-N-SH cells treated with 100 IU/ml IFNα at 16 h pre- or 1.5, 3 or 6 h post-infection to induce an antiviral state were infected with the different TBEV strains for 26 h at MOI of 1. The number of infectious virus produced after one round of infection in A549 cells is shown in Fig. 6A, while the results normalized to mock treated, yet infected cells are shown in Fig. 6B. Production of infectious progeny was largely inhibited for all viruses when exogenous IFNα was added pre-infection (3–4 logs, Fig. 6A,B -16 h). The inhibiting effects of exogenous IFNα were much smaller when added post TBEV infection and decreased over time (Fig. 6A,B 1.5–6 h), suggesting that all TBEV strains could interfere with the IFN-I signaling cascade as soon as infection had been initiated. Any differences in (inhibition of) infectious virus production between the different TBEV strains were not determined to be significant by Mann–Whitney test.

Results of the equivalent experiment performed in the neuroblastoma cell line SK-N-SH are shown in Fig. 6C,D. Pre-treatment with 100 IU/ml IFNα inhibited infection of all strains over 3 logs (Fig. 6C), while addition of IFNα after TBEV infection inhibited production of infectious virus to a lesser extent (maximal 1.6 logs; Fig. 6C,D). Although infectious progeny production of Neudoerfl appeared less affected by IFNα treatment than progeny production of Hypr and Salland (Fig. 6D), observed differences were not significant (Mann–Whitney test).

## Discussion

Here, we report the successful rescue of the divergent TBEV-Eu strain Salland using the ISA technology and its subsequent phenotypic characterization in vitro. Assessment of growth kinetics on respiratory and brain resident cell lines, infection studies using human moDCs, as well as analysis of viral interactions with the IFN-I system, revealed that the novel TBEV strain Salland mimics the highly pathogenic Hypr rather than to the mildly pathogenic Neudoerfl. Overall, our study described the first characterization of novel, divergent TBEV-Eu strains recently detected in both the Netherlands and the United Kingdom.

By in vitro comparison of the TBEV-Eu strain Salland with two TBEV-Eu strains of known pathogenicity, we aimed to get a better insight into its pathogenic potential in humans. On the basis of the observation that Salland behaves more alike the highly pathogenic TBEV-Eu strain Hypr than the mildly pathogenic TBEV-Eu strain Neudoerfl in both peripheral and brain-resident cell lines, while no differences between the strains were observed in moDCs, we hypothesize that Salland is a TBEV-Eu strain with pathogenic potential. Evidently, caution needs to be taken when extrapolating in vitro data to the situation in vivo. Yet, multiple studies have previously shown that TBEV replication efficiency in vitro is often associated with pathogenicity in vivo^29,30,33,35,47,48^, with highly pathogenic strains replicating faster and to higher titers than mildly pathogenic strains in a multitude of cell lines^29,30,35,47,48^ and human whole blood samples infected ex vivo^49^. Nonetheless, reports in which TBEV growth kinetics in vitro do not directly correlate with pathogenicity in vivo are also known. For example, two natural strains showing similar replication kinetics in vitro, differed in their neuroinvasiveness in vivo, which was hypothesized to be due to different interactions with the host immune response^6^. Additionally, a cell culture adapted strain that showed enhanced replication in vitro, was found to be impaired in vivo^50^ and a TBEV strain with a synthetically elongated 3′-UTR which was impaired in vitro, but highly pathogenic in vivo^51^. Consequently, even though found no differences in the interactions with the IFN-I system were observed for the TBEV strains used in our study and the strains used were neither cell-culture adapted nor mutated, additional in vivo data is needed to confirm the pathogenic potential of the novel TBEV-Eu strain Salland.

With respect to pathogenicity of the divergent TBEV-Eu strain Salland, it is of interest to note that multiple human TBE cases have already been reported in the area where the TBEV-Eu strain Salland was originally detected. To date, we have not been able to sequence nor isolate virus from TBE patients infected in this area. However, as no TBEV strains other than ‘Salland’ have been detected in this area to date, this observation serves as indirect evidence that the TBEV-Eu strain Salland is indeed pathogenic in humans.

The IFN-I response is crucial to control and restrict TBEV replication both in vitro and in vivo. In absence of a properly functioning IFN-I response, mice infected with either TBEV or the closely related flavivirus Langat virus (LGTV) succumb earlier to infection, with more extensive viral replication in peripheral organs and rapid neuro-invasion^41,42^. As TBEV has been reported to circumvent the IFN-I response both passively^29,44^ and actively^52–55^ and as for other neurotropic (flavi)viruses a greater ability to evade the IFN-I response in vitro had previously been associated with high pathogenicity in vivo^45,46,56,57^, we examined the interactions with the IFN-I response for the different TBEV strains used in this study. Our results indicate that all three TBEV strains are able to interfere with the IFN-I signaling cascade to a similar extent, even though two out of three strains used in this study (Neudoerfl and Hypr) are reported to differ in pathogenic potential. IFN-I antagonism of flaviviruses, including TBEV, has been attributed to three non-continuous amino acid regions of NS5^55^. Retrospective analysis revealed that these regions are highly conserved between the TBEV strains in this study (data not shown), corroborating our phenotypic results. While differences in stimulation of the IFN-I response were observed in two out of three cell types assessed, these were most likely the result of differences in replication kinetics. When IFN-I induction was normalized for replication in A549 and SK-N-SH and in moDCs, a cell type in which all viruses grew to comparable titers, no differences in IFN induction could be observed. Overall, our data suggests that (putative) differences in pathogenicity between the strains employed are not (directly) caused by differential interactions with the IFN-I response.

Factors contributing to pathogenicity of specific TBEV strains are still poorly understood. Next to evasion of the IFN-I response, pathogenicity has been linked to suppression of immune cell function^49^ and deletions or poly(A) insertions in the variable region of the 3′UTR^58,59^. Interestingly, the 3′-UTR length of the different TBEV subtypes appears to be inversely correlated to their pathogenic potential^60^. Furthermore, the 3′-UTR of TBEV strains isolated from severely ill patients often contain deletions in their variable region^58,61^. Compared to the 3′-UTR of Neudoerfl (764 nucelotides), the 3′-UTR of both Salland and Hypr (536 and 458 nucleotides, respectively) are relatively short. Future studies are needed to address whether this difference in 3′-UTR length may contribute to the phenotypic differences observed in vitro.

One of the limitations of the current study is that the TBEV-Eu strain characterized was not isolated from ticks nor patient material, but instead rescued based on a close-to-complete genome. Whole genome sequencing of the TBEV-Eu strain Salland left respectively 7 and 2 nucleotides at the extreme ends of the 5′- and 3′-UTR unresolved. As these nucleotides are highly conserved in viruses of the TBE serocomplex (including the different TBEV subtypes, but also LIV, Omsk hemorrhagic fever virus (OHFV) and Langat virus (LGTV)), it was considered justified to complete the genome by adding these sequences in silico*.* It should nevertheless be noted that correctness of the final nucleotides of the 5′- and 3′-UTRs for our and other TBEV strains needs to be confirmed in the future with more adequate approaches. Infectious Subgenomic Amplicons were previously successfully used to generate ‘wild-type’ TBEV infectious particles^32,62^ and were shown to have potential for isolation of viruses that cannot be directly cultured from clinical and animal samples^63^. Considering the high probability that the complete genome sequence of the TBEV-Eu strain detected in ticks and the rescued virus are identical, the only difference expected between the rescued virus and an isolate are viral quasispecies that may be present in ticks, but will evidently be missed upon use of reverse genetic techniques.

Additionally, the cell lines and types used in this study may not fully resemble the TBEV target cells in vivo*.* Under natural circumstances, TBEV is considered to initially infect DCs, most likely Langerhans cells, at the primary site of infection. Subsequently, TBEV causes viremia and replicates in peripheral organs. Finally, TBEV might invade the CNS through mechanisms that are still incompletely understood. In the CNS, neurons seem to be the main target cells of TBEV^7,64,65^. Being often used in TBEV pathogenicity studies^29,35,47,66,67^, the cell types used in this study served as suitable and convenient models for initial characterization of the TBEV-Eu strain Salland. To confirm the findings obtained in this study, further studies using more differentiated cell cultures in vitro, including neurons and Langerhans cells, and in vivo infection experiments need to be performed.

In conclusion, in the current study we described the rescue of the TBEV-Eu strain Salland by use of Infectious Subgenomic Amplicons, a technique previously described by Aubry and co-workers^32^. For TBEV, the ISA method was previously successfully used to generate ‘wild-type’ virus particles^32,62,68^, to engineer attenuated strains with a potential use as live attenuated vaccines^62^, to study drug resistance mutations^69^, and to rescue inactivated virus from an animal sample^63^. Here, the ISA method proofed its worth for phenotypic characterization of a novel TBEV variant for which a (near-complete) full genome sequence was available, but for which isolation could not be attempted. Our data suggests that the novel, divergent TBEV-Eu strain Salland may have pathogenic potential. The TBEV-Eu strain Salland described in this study will be made available to the scientific community via European Virus Archive-GLOBAL (EVAg).

## Materials and methods

### Phylogenetic inference

To analyze the phylogenetic relationship of the TBEV sequences obtained from ticks in the Netherlands (GenBank accession numbers LC171402 and MH021184)^21,22^ with known TBEV-Eu strains, all TBEV-Eu sequences encompassing the complete coding sequence were selected from GenBank (n = 88, retrieved on September 18, 2021). Multiplicities, synthetic and clonal sequences (n = 11) were omitted, while the reference sequence of LIV (NC_001809), a tick-borne flavivirus that is phylogenetically more closely related to TBEV-Eu than TBEV-FE and TBEV-Sib are^19^ and that is primarily found in the British Isles, was included as an outgroup. The remaining 78 sequences were aligned in Jalview^70^ using the Mafft algorithm, after which the 5′-and 3′-UTRs were removed. The IQTree web server^71^ was used to select the best-fit evolutionary model (GTR + F + R3) and infer a maximum likelihood (ML) tree. Robustness was assessed by ultrafast bootstrapping using 1000 replicates. The eventual ML tree was visualized and edited using FigTree^72^.

### Ticks

Ticks were caught in spring 2016 by flagging at the same location in National Park ‘Sallandse Heuvelrug’ where TBEV-positive ticks were detected in 2015^21^. Ticks were pooled in 600 µl GLY medium (MediaProducts BV, Groningen, the Netherlands) and disrupted by lysis matrix Z tissue homogenization beads (MP biomedicals, Santa Ana, CA, USA). 200 µl of the tick homogenate was added to 250 µl MagNA Pure Lysis buffer and total nucleic acids were extracted using the MagNA Pure 96 Instrument (Roche). qRT-PCR was performed using the pan-flavivirus prime/probe set described previously by Patel and coworkers^73^ in combination with the TaqMan Fast Virus 1-Step Master Mix (Thermo Fisher Scientific, Waltham, MA, USA). The remaining tick homogenate was supplemented with 10% FCS and stored at − 80 °C for virus isolation.

### Illumina MiSeq sequencing

Amplicons previously used to sequence the TBEV-NL genome by Sanger sequencing (GenBank accession number LC171402) and RNA isolated from viral culture supernatants were sequenced using the Illumina MiSeq instrument (Illumina, San Diego, CA, USA). Viral RNA was transcribed into cDNA using the SuperScript III reverse transcriptase (Invitrogen) with random nonamers (New England Biolabs), followed by second strand DNA synthesis using NEBNext Second Strand Synthesis Module (New England Biolabs). dsDNA was concentrated using the DNA Clean & Concentrator™-5 kit (Zymo research) according to manufacturer’s instructions. Library preparation was performed using Nextera XT DNA library preparation kit (Illumina, San Diego, CA, USA), MiSeq PE300 sequencing (Illumina, San Diego, CA, USA) and demultiplexing of raw sequence data were all outsourced to BaseClear B.V. (Leiden, the Netherlands). The CLC Genomics workbench (CLCbio, Aarhus, Denmark) was used to trim the reads. Subsequently, all possible human reads were removed, remaining reads were mapped against the TBEV-EU reference genome (GenBank accession number NC_001672) and a consensus sequence was extracted.

### Cells

The baby hamster kidney fibroblast cell line BHK-21 (ATCC CCL-10) were maintained in RPMI-1640 (Gibco™, Thermo Fisher Scientific, Waltham, MA, USA) supplemented with 10% Fetal Calf Serum (FCS; Biowest, Nuaillé, France), 100 U/ml penicillin and 100 µg/ml streptomycin (Biowest, Nuaillé, France). The human lung epithelial cell line A549 (ATCC CCL-185), the human neuroblastoma cell line SK-N-SH (ATCC HTB-11), the human glioblastoma cell lines U87-MG (ATCC HTB-14) and T98G (ATCC CRL-1690) and the human oligodendrocytic cell line MO3.13 were all maintained in Dulbecco’s Modified Eagle’s MEM (DMEM; Gibco™, Thermo Fisher Scientific, Waltham, MA, USA) supplemented with 10% FCS (FCS; Biowest, Nuaillé, France), 100 units/ml penicillin and 100 µg/ml streptomycin (Biowest, Nuaillé, France). Finally, green monkey kidney cell line Vero-E6 (ATCC CRL-1586) were maintained in DMEM (Gibco™, Thermo Fisher Scientific, Waltham, MA, USA) supplemented with 5% FCS (Biowest, Nuaillé, France), 100 units/ml penicillin and 100 µg/ml streptomycin (Biowest, Nuaillé, France). All cells were cultured at 37 °C under 5% CO_2_.

### Viruses

The TBEV-Eu reference strain Neudoerfl was obtained via the European Virus Archive (EVAg, catalog number 003v-EVA471). Viral working stocks were obtained by passaging the virus twice on Vero-E6 cells at an MOI of 0.01 and harvesting 5 days post infection.

TBEV-Eu strains Salland (Genbank accession number BK061374) and Hypr (Genbank accession number U39292) were produced using the ISA technique as previously described by Aubry and coworkers in 2014^32^. Briefly, a dsDNA sequence was constructed in which the complete TBEV genome was flanked by the human cytomegalovirus promoter (pCMV) at the 5′-end and the hepatitis delta ribozyme followed by the simian virus 40 polyadenylation signal (HDR/SV40pA) at the 3′-end. The designed dsDNA was divided into three individual ‘amplicons’ of 3–4 kb including an overlap of 80–120 nucleotides with the adjoining amplicons. Individual amplicon sequences flanked by DraI restriction sites were synthesized at BaseClear (Leiden, the Netherlands) and delivered in a standard plasmid vector. Amplicons were linearized by DraI digestion, purified from gel and subsequently electroporated into BHK-21 cells at equimolar ratios. Cell culture medium was changed 1 day post transfection, and supernatants were harvested upon detection of cytopathic effect (5 to 6 days post transfection). Viral working stocks were created by passaging the virus once on Vero-E6 at an MOI of 0.01.

### Virus growth curves and titrations

Growth kinetics of the different TBEV strains were determined by infecting cells at a low MOI (0.01) in DMEM (Gibco™, Thermo Fisher Scientific, Waltham, MA, USA) supplemented with 2% FCS (Biowest, Nuaillé, France), 100 units/ml penicillin and 100 µg/ml streptomycin (Biowest, Nuaillé, France). Unbound virus was washed away at 2 h post infection, after which cells were maintained in DMEM supplemented with 10% FCS, 100 units/ml penicillin, 10 µg/ml streptomycin and 25 mM HEPES (Gibco™, Thermo Fisher Scientific, Waltham, MA, USA). Virus cultures were sampled every 24 h until 4 days post infection. Production of infectious virus was determined by plaque assay. Briefly, monolayers of A549 cells seeded in 12 wells cluster plates were infected with 10× serial dilutions of virus for 1.5 h. Cells were overlaid with 1× minimal essential medium (Gibco™, Thermo Fisher Scientific, Waltham, MA, USA) containing 2% fetal bovine serum (Biowest, Nuaillé, France), 25 mM HEPES (Gibco™, Thermo Fisher Scientific, Waltham, MA, USA), 100 units/ml penicillin and 100 µg/ml streptomycin (Biowest, Nuaillé, France) and 1% SeaPlaque GTG agarose (Lonza, Basel, Switzerland) and incubated for 5 days at 37 °C under 5% CO_2_. The cells were then fixed with 10% formaldehyde and stained with crystal violet (1% [w/v] in 20% ethanol) to visualize plaques. Plaques were counted and expressed as plaque-forming units per ml (PFU/ml). Genome equivalents per ml (GE/ml) were determined by qRT-PCR using the pan-TBEV primer/probe set previously described by Schwaiger and Casssinotti^74^ in combination with the TaqMan Fast Virus 1-Step Master Mix (Thermo Fisher Scientific, Waltham, MA, USA). A 10× dilution range of linearized plasmid encoding the TBEV 3′UTR (ranging from 2.5 × 10^8^ to 2.5 × 10^2^ genome copies) was used as a standard curve.

### Cell viability assays

Cell viability was assessed using PrestoBlue reagent (Invitrogen™, Thermo Fisher Scientific, Waltham, MA, USA) with minor adaptations to manufacturer’s instructions. Briefly, A549 or SK-N-SH cells were seeded into 96-well cluster plates and infected with the different TBEV strains for 72 or 96 h as described above. Cell culture medium was replaced for complete cell culture medium including 1× PrestoBlue reagent. Plates were incubated for 4 h at 37 °C under 5% CO_2_. Next, plates were sealed and absorbance was read at 570 nm using 600 nm as a reference in a Multiskan™ FC (Thermo Fisher Scientific, Waltham, MA, USA) microplate reader. All measurements were performed in eightfold.

### Infection of monocyte-derived dendritic cells (moDCs)

Peripheral blood mononuclear cells (PBMCs) were obtained from healthy volunteers at the National Institute for Public Health and the Environment (RIVM, the Netherlands). Blood was collected in heparin tubes and the mononuclear fraction was isolated by density gradient centrifugation (Lymphoprep, Nycomed, Zürich, Switzerland). Monocytes were purified by positive selection using magnetic human CD14 microbeads (Miltenyi Biotec, Bergisch Gladbach, Germany). Purity of the isolated monocytes was confirmed to be > 95% using flow cytometry. Differentiation into monocyte-derived dendritic cells (moDCs) was induced by culturing for 7 days in Iscove’s Modified Dulbecco’s Medium (IMDM, Gibco™, Thermo Fisher Scientific, Waltham, MA, USA) supplemented with 1% heat-inactivated fetal bovine serum (HyClone, Logan, UT, USA), 1× penicillin/streptomycin/glutamine (Gibco™, Thermo Fisher Scientific, Waltham, MA, USA), 500 U/mL human GM-CSF (PeproTech, Rocky Hill, NJ, USA), and 800 U/mL human IL-4 (Active BioScience, Hamburg, Germany). After 7 days of culture, moDCs were infected with the different TBEV strains at MOIs of 0.1, 1 and 10. At 26 hpi, a time point selected to obtain maximal virus yield produced in one infectious cycle^75^, supernatants were harvested for virus titration. Additionally, cells were fixed using 4% PFA, permeabilized using 0.02% Triton X-100 in PBS and stained using mouse anti-flavivirus antibody 4G2 (1:500; Merck, Darmstadt, Germany) followed by goat anti-mouse-AlexaFluor488 (1:200; Abcam, Cambridge, United Kingdom). Sytox Orange staining (167 nM; Thermo Fisher Scientific, Waltham, MA, USA) was added to stain the nuclei. Cells were imaged using a Leica DMIL LED fluorescence microscope (Leica, Wetzlar, Germany). To quantify the number infected cells, snapshots of both the Sytox Orange stained nuclei and AF488-stained TBEV-infected cells were taken of 10 randomly selected nuclei-containing fields per well, after which the number of nuclei and infected cells were counted by eye. An example of the Sytox Orange/4G2-stained moDC staining is shown in Fig. S4.

### Type I interferon assays

The amount of type I interferon produced upon infection of A549, SK-N-SH and moDCs was determined using HEK-Blue™ IFN-α/β Cells (InvivoGen, San Diego, CA, USA) according to manufacturer’s instructions. Shortly, 20 µl of supernatant was UV inactivated for 15 min, mixed with 50,000 HEK-Blue™ IFN-α/β cells in a 96-wells cluster, and incubated overnight at 37 °C under 5% CO_2_. The next day, 20 µl HEK-Blue supernatant was transferred to 180 µl pre-warmed QuantiBlue, samples were incubated for 2 h after which the colorimetric reaction was measured at 620 nm.

To determine the sensitivity of the different TBEV strains to type I interferons, exogenous recombinant human IFN-alpha 2a (100 U/ml; R&D Systems, MN, USA) was added to A549 and SK-N-SH cell cultures at different time points pre- and post-infection (MOI 1). At 26 h post infection, supernatants were harvested for titration.

### Ethics statement

This study was conducted according to the principles described in the Declaration of Helsinki and for the collection of samples and subsequent analyses, all blood donors provided written informed consent. Blood samples were processed anonymously and the research goal, primary cell isolation, required no review by an accredited Medical Research Ethics Committee (MREC), as determined by the Dutch Central Committee on Research involving human subjects (CCMO).

## Supplementary Information

Supplementary Information.

## Data Availability

Viral sequences obtained or designed in this study are openly available in GenBank at https://www.ncbi.nlm.nih.gov/genbank/ under reference numbers ON502378 and BK061374. All other relevant data are within the manuscript and its Supporting Information files.
