# Supplementary material for: Rescue and in vitro characterization of a divergent TBEV-Eu strain from the Netherlands
Source: Sci Rep. 2023 Feb 18;13:2872. doi: 10.1038/s41598-023-29075-0 (PMC9938877; doi:10.1038/s41598-023-29075-0)
Supplement: Supplementary file 1 — Supplementary Information. [file 41598_2023_29075_MOESM1_ESM.pdf]

Supplementary information

Supplementary Materials and Methods

Analysis of the extreme ends of the tick-borne flavivirus 5'- and 3'-UTR

For comparison of the 5'-UTRs of TBEV-Eu and closely related tick-borne flaviviruses, a BLAST search was performed (On October 10, 2021) using the 132 nucleotide 5'-UTR of the TBEV reference sequence (GenBank accession number NC_001672) and all sequences with a coverage of 100% were retrieved. Sequences (n= 12) that were of synthetic origin were omitted, after which the remaining 5'-UTR sequences were aligned in Jalview[1] using the Mafft algorithm. The first seven nucleotides of the 5'-UTRs were inspected for nucleotide polymorphisms by eye.

As the 3'-UTR is known to be much more variable than the 5'-UTR, only the final 100 nucleotides of the 3'-UTR of the TBEV reference sequence (GenBank accession number NC_001672) were subjected to a BLAST search. All sequences with a coverage of ≥ 90% were retrieved (October 10, 2021), sequences of synthetic origin were omitted, and the remaining sequences were aligned in Jalview[1] using the Mafft algorithm. Sequences with incomplete coverage of the final 10 nucleotides were removed, as it could not be excluded that the final nucleotides were missing due to incomplete sequencing of the genome. The extreme ends of the 3'-UTR were inspected for nucleotide polymorphisms by eye.

Supplementary references

1 Waterhouse, A. M., Procter, J. B., Martin, D. M., Clamp, M. & Barton, G. J. Jalview Version 2--a multiple sequence alignment editor and analysis workbench. *Bioinformatics* **25**, 1189-1191, doi:10.1093/bioinformatics/btp033 (2009).

Extreme end of 5'-UTR (nucleotide position 5' → 3') 1	2	3	4	5	6	7	# of sequences	GenBank accession #	Tick-borne flavivirus subtype	Host	Region
**A**	**G**	**A**	**T**	**T**	**T**	**T**	187	various	various[2]	various	various
.	.	.	.	.	**C**	.	2	KP331441<br>KP331441	TBEV-Eu	*Ixodes persulcatus*	Eastern Siberia
.	.	.	.	**A**	.	.	2	EU715143<br>EU715154	TBEV-Sib	*Ixodes persulcatus*	Western Siberia
.	.	**T**	.	.	.	.	1	EU715156	TBEV-Sib	*Ixodes persulcatus*	Western Siberia

[1] nucleotide position based on the TBEV reference sequence (GenBank accession number NC_001672) [2] including TBEV-Eu, TBEV-Sib, TBEV-FE, TBEV-Him, TBEV-Bkl, LIV and SGEV

Table S1: Comparison of the extreme ends of the 5'-UTR between different tick-borne flaviviruses. Full length 5'-UTR sequences of 192 tick-borne flavivirus strains were retrieved from GenBank and aligned in Jalview using the Mafft algorithm. Nucleotides were color coded and polymorphisms in the first seven nucleotides of the 5'-UTR were assessed by eye.

Extreme end of 3'-UTR (nucleotide position 5' → 3')[1] 11134	11135	11136	11137	11138	11139	11140	11141	# of sequences	GenBank accession #	Tick-borne flavivirus subtype	Host	Region
**C**	**A**	**C**	**C**	**C**	**G**	**C**	**T**	180	various	various[2]	various	various
.	**C**	.	.	.	.	.	.	2	HQ901366<br>HQ901367	TBEV-FE	*Homo sapiens*	Russian Far East

[1] nucleotide position based on the TBEV reference sequence (GenBank accession number NC_001672) [2] including TBEV-Eu, TBEV-Sib, TBEV-FE, TBEV-Him, TBEV-Bkl, LIV, SGEV, LGTV and OHFV

Table S2: Comparison of the extreme ends of the 3'-UTR between different tick-borne flaviviruses. The final 100 nucleotides of the 3'-UTR of 182 tick-borne flaviviruses were retrieved from GenBank and aligned in Jalview using the Mafft algorithm. Nucleotides were color coded and polymorphisms in the final eight nucleotides of the 5'-UTR were assessed by eye. Indicated nucleotide position was based on the TBEV reference sequence (GenBank accession number NC_001672).

Anchored capsid/core protein (C; 1-112) with cleaved capsid/core indicated in grey – 4 amino acid changes (3,57% difference)

```
Neudoerfl      MVKKAILKGKGGGPPRRVSKETATKTRQPRVQMPNGLVLMRMMGILWHAVAGTARNPVLK   60
Salland        MVRKAILKGKGGGPPRRASKETAAKTRQPRVQMPNGLVLMRMMGILWHAVAGTARNPVLK   60
               **:**************.*****:************************************

Neudoerfl      AFWNSVPLKQATAALRKIKRTVSALMVGLQKRGKRRSATDWMSWLLVITLLG           112
Salland        AFWNSVPLKQATAALRKIKRTVSALMVGLQKRGKRRSATDWMSWLLVIILLG           112
               ************************************************ ***
```

preM protein (113-280), with mature M protein in grey – 6 amino acid changes (3,57% difference)

```
Neudoerfl      MTLAATVRKERDGSTVIRAEGKDAATQVRVENGTCVILATDMGSWCDDSLSYECVTIDQG   172
Salland        MTLAATVRKEMDGTTVIRAEGKDAATQVRVENGTCVILATDMGSWCDDSLSYECVTIDQG   172
               ********** **:**********************************************

Neudoerfl      EEPVDVDCFCRNVDGVYLEYGRCGKQEGSRTRRSVLIPSHAQGELTGRGHKWLEGDSLRT   232
Salland        EEPVDVDCFCRNVDGVYLEYGRCGKQEGSRTRRSVLIPSHAQGELTGRGHKWLEGDSLRT   232
               ************************************************************

Neudoerfl      HLTRVEGWVWKNKLLALAMVTVVWLTLESVVTRVAVLVVLLCLAPVYA               280
Salland        HLTRVEGWVWKNRLLALAMVAVVWLTLESVLTRVVVLVVLLCLAPVYA               280
               ************:*******:*********:***.*************
```

Envelope (E) protein (281-776) – 5 amino acid changes (1,01% difference)

```
Neudoerfl      SRCTHLENRDFVTGTQGTTRVTLVLELGGCVTITAEGKPSMDVWLDAIYQENPAKTREYC   340
Salland        SRCTHLENRDFVTGTQGTTRVTLVLELGGCVTITAEGKPSMDVWLDAIYQENPAKTREYC   340
               ************************************************************

Neudoerfl      LHAKLSDTKVAARCPTMGPATLAEEHQGGTVCKRDQSDRGWGNHCGLFGKGSIVACVKAA   400
Salland        LHAKLSDTKVAARCPTMGPATLAEEHQGGTVCKRDQSDRGWGNHCGLFGKGSIVACVKAS   400
               ***********************************************************:

Neudoerfl      CEAKKKATGHVYDANKIVYTVKVEPHTGDYVAANETHSGRKTASFTISSEKTILTMGEYG   460
Salland        CEAKKKATGHVYDANKIVYTVKVEPHTGDYVAANETHSGRKTASFTVSSEKTILTMGEYG   460
               **********************************************:*************

Neudoerfl      DVSLLCRVASGVDLAQTVILELDKTVEHLPTAWQVHRDWFNDLALPWKHEGAQNWNNAER   520
Salland        DVSLLCRVASGVDLAQTVILELDKTVEHLPTAWQVHRDWFNDLALPWKHEGAQNWNNAER   520
               ************************************************************

Neudoerfl      LVEFGAPHAVKMDVYNLGDQTGVLLKALAGVPVAHIEGTKYHLKSGHVTCEVGLEKLKMK   580
Salland        LVEFGAPHAVKMDVYNLGDQTGVLLKALAGVPVAHIEGTKYHLKSGHVTCEVGLEKLKMK   580
               ************************************************************

Neudoerfl      GLTYTMCDKTKFTWKRAPTDSGHDTVVMEVTFSGTKPCRIPVRAVAHGSPDVNVAMLITP   640
Salland        GLTYTMCDKTKFTWKRAPTDSGHDTVVMEVTFSGTKPCRIPVRAVAHGSPDVNVAMLITP   640
               ************************************************************

Neudoerfl      NPTIENNGGGFIEMQLPPGDNIIYVGELSHQWFQKGSSIGRVFQKTKKGIERLTVIGEHA   700
Salland        NPTIENNGGGFIEMQLPPGDNIIYVGDLSHQWFQKGSSIGRVFQKTRKGIERLTVIGEHA   700
               **************************:*******************:*************

Neudoerfl      WDFGSAGGFLSSIGKAVHTVLGGAFNSIFGGVGFLPKLLLGVALAWLGLNMRNPTMSMSF   760
Salland        WDFGSAGGFLNSIGKAVHTVLGGAFNSIFGGVGFLPKLLLGVALAWLGLNMRNPTMSMSF   760
               **********.*************************************************

Neudoerfl      LLAGGLVLAMTLGVGA    776
Salland        LLAGGLVLAMTLGVGA    776
               ****************
```

Non-structural protein NS1 (777-1128) – 12 amino acid changes (3,41% difference)

```
Neudoerfl     DVGCAVDTERMELRCGEGLVVWREVSEWYDNYAYYPETPGALASAIKETFEEGSCGVVPQ   836
Salland       DVGCAVDTERMELRCGEGLVVWREVSEWYDNYAYYPETPGALASAIKETFEEGSCGIVPQ   836
              ********************************************************:***

Neudoerfl     NRLEMAMWRSSVTELNLALAEGEANLTVVVDKFDPTDYRGGVPGLLKKGKDIKVSWKSWG   896
Salland       NRLEMAMWRSSVTELNLALAEGDANLTVVVDKFDPTDYRGGIPGLLKKGKDITVSWKAWG   896
              **********************:******************:**********.****:**

Neudoerfl     HSMIWSIPEAPRRFMVGTEGQSECPLERRKTGVFTVAEFGVGLRTKVFLDFRQEPTHECD   956
Salland       HSMIWSIPEAPRRFMVGTEGQGECPLEKRKTGVFTVAEFGVGLRTKVFLDFRQESTHECD   956
              *********************.*****:************************** *****

Neudoerfl     TGVMGAAVKNGMAIHTDQSLWMRSMKNDTGTYIVELLVTDLRNCSWPASHTIDNADVVDS   1016
Salland       TGVMGAAVKNGMAVHTDQSLWMKSMKNETGTYIVELLVTDLRNCSWPASHTIDNADVVDS   1016
              *************:********:****:********************************

Neudoerfl     ELFLPASLAGPRSWYNRIPGYSEQVKGPWKYTPIRVIREECPGTTVTINAKCDKRGASVR   1076
Salland       ELFLPASLAGPRSWYNRIPGYSEQVKGPWKYTPIRVIREECPGTTVTINAKCDKRGASVR   1076
              ************************************************************

Neudoerfl     STTESGKVIPEWCCRACTMPPVTFRTGTDCWYAMEIRPVHDQGGLVRSMVVA           1128
Salland       STTESGKVIPEWCCRACTMPPVTFRTGTDCWYAMEIRPVHAQGGLVRSMVVA           1128
              **************************************** ***********
```

Non-structural protein NS2a (1129-1358) – 9 amino acid changes (3,91% difference)

```
Neudoerfl     DNGELLSEGGVPGIVALFVVLEYIIRRRPSTGTTVVWGGIVVLALLVTGMVRIESLVRYV   1188
Salland       DNGELLSEGGVPGIVALFVVLEYIIRRRPSTGTTVVWGGMVVLALLVTGMVKIESLVRYV   1188
              ***************************************:***********:********

Neudoerfl     VAVGITFHLELGPEIVALMLLQAVFELRVGLLSAFALRRSLTVREMVTTYFLLLVLELGL   1248
Salland       VAVGITFHLELGPEIVALMLLQAVFELRVGLLSAFALRRSLTVREMVTTYFLLLVLELGL   1248
              ************************************************************

Neudoerfl     PGASLEEFWKWGDALAMGALIFRACTAEGKTGAGLLLMALMTQQDVVTVHHGLVCFLSVA   1308
Salland       PGASFEDFWKWGDALAMGALMFRACTAEGKTGAGLLLMALMTQQDVVTVHHGLVCFLSVA   1308
              ****:*:*************:***************************************

Neudoerfl     SACSVWRLLKGHREQKGLTWVVPLAGLLGGEGSGIRLLAFWELSAHRGRR             1358
Salland       SACSVWRLLRGHREQKGLTWIVPLVGLLGGEGSGIRLLAFWELAAHRGRR             1358
              *********:**********:***.******************:******
```

Non-structural protein NS2b (1359-1489) – 5 amino acid changes (3,82% difference)

```
Neudoerfl     SFSEPLTVVGVMLTLASGMMRHTSQEALCALAVASFLLLMLVLGTRKMQLVAEWSGCVEW   1418
Salland       SFSEPLTVVGVMLTLASGMMRHTSQEALCALAVASFLLLMLVLGTRKMQLVAEWSGCVEW   1418
              ************************************************************

Neudoerfl     YPELVNEGGEVSLRVRQDAMGNFHLTELEKEERMMAFWLIAGLAASAIHWSGILGVMGLW   1478
Salland       HPELVNEGGEVSLRVRQDSMGNFHLTELEKEERMMAFWLLAGLVASAFHWSGILGVMGLW   1478
              :*****************:********************:***.***:************

Neudoerfl     TLTEMLRSSRR                                                    1489
Salland       TLTEMLRSSRR                                                    1489
              ***********
```

Non-structural protein NS3 (1490-2110) – 8 amino acid changes (1,29% difference)

```
Neudoerfl      SDLVFSGQGGRERGDRPFEVKDGVYRIFSPGLFWGQNQVGVGYGSKGVLHTMWHVTRGAA   1549
Salland        SDLVFSGQGGRERGDRPFEVKDGVYRIFSPGLLWGQNQVGVGYGSKGVLHTMWHVTRGAA   1549
               ********************************:***************************

Neudoerfl      LSIDDAVAGPYWADVREDVVCYGGAWSLEEKWKGETVQVHAFPPGRAHEVHQCQPGELIL   1609
Salland        LSIDDAVAGPYWADVREDVVCYGGAWSLEEKWKGETVQVHAFPPGKAHEVHQCQPGELIL   1609
               *********************************************:**************

Neudoerfl      DTGRKLGAIPIDLVKGTSGSPILNAQGVVVGLYGNGLKTNETYVSSIAQGEAEKSRPNLP   1669
Salland        DTGKKLGAIPIDLVKGTSGSPILNAQGVVVGLYGNGLKTNETYVSSIAQGEAEKSRPNLP   1669
               ***:********************************************************

Neudoerfl      QAVVGTGWTSKGQITVLDMHPGSGKTHRVLPELIRQCIDRRLRTLVLAPTRVVLKEMERA   1729
Salland        QAVVGTGWTSKGQITVLDMHPGSGKTHRVLPELIRQCIDRRLRTLVLAPTRVVLKEMERA   1729
               ************************************************************

Neudoerfl      LNGKRVRFHSPAVSDQQAGGAIVDVMCHATYVNRRLLPQGRQNWEVAIMDEAHWTDPHSI   1789
Salland        LNGKRVKFHSPAVSDQQAGGAIVDVMCHATYVNRRLLPQGRQNWEVAIMDEAHWTDPHSI   1789
               ******:*****************************************************

Neudoerfl      AARGHLYTLAKENKCALVLMTATPPGKSEPFPESNGAITSEERQIPDGEWRDGFDWITEY   1849
Salland        AARGHLYTLAKENKCALVLMTATPPGKNEPFPESNGAITSEERQIPDGEWRDGFDWITEY   1849
               ***************************.********************************

Neudoerfl      EGRTAWFVPSIAKGGAIARTLRQKGKSVICLNSKTFEKDYSRVRDEKPDFVVTTDISEMG   1909
Salland        EGRTAWFVPSIAKGGVIARTLRQKGKSVICLNSKTFEKDYSRVRDEKPDFVVTTDISEMG   1909
               ***************.********************************************

Neudoerfl      ANLDVSRVIDGRTNIKPEEVDGKVELTGTRRVTTASAAQRRGRVGRQDGRTDEYIYSGQC   1969
Salland        ANLDVSRVIDGRTNIKPEEVDGKVELTGTRRVTTASAAQRRGRVGRQDGRTDEYIYSGQC   1969
               ************************************************************

Neudoerfl      DDDDSGLVQWKEAQILLDNITTLRGPVATFYGPEQDKMPEVAGHFRLTEEKRKHFRHLLT   2029
Salland        DDDDSGLVQWKEAQILLDNITTLRGPVATFYGPEQDKMPEVAGHFRLTEEKRKHFRHLLT   2029
               ************************************************************

Neudoerfl      HCDFTPWLAWHVAANVSSVTDRSWTWEGPEANAVDEASGDLVTFRSPNGAERTLRPVWKD   2089
Salland        HCDFTPWLAWHVAANVSSVTDRSWTWEGPEANAVDEASGDLVTFRSPNGAERTLRPVWRD   2089
               **********************************************************:*

Neudoerfl      ARMFKEGRDIKEFVAYASGRR                                          2110
Salland        ARMFREGRDIKEFVAYASGRR                                          2110
               ****:****************
```

Non-structural protein NS4a (2111-2236)- 1 amino acid change (0,79% difference)

```
Neudoerfl      SFGDVLTGMSGVPELLRHRCVSALDVFYTLMHEEPGSRAMRMAERDAPEAFLTMVEMMVL   2170
Salland        SFGDVLTGMSGVPELLRHRCVSALDVFYTLMHEEPGSRAMRMAERDAPEAFLTVVEMMVL   2170
               *****************************************************:******

Neudoerfl      GLATLGVIWCFVVRTSISRMMLGTLVLLASLLLLWAGGVGYGNMAGVALIFYTLLTVLQP   2230
Salland        GLATLGVIWCFVVRTSISRMMLGTLVLLASLLLLWAGGVGYGNMAGVALIFYTLLTVLQP   2230
               ************************************************************

Neudoerfl      EAGKQR                                                         2236
Salland        EAGKQR                                                         2236
               ******
```

2K protein (2237-2259) – 0 amino acid changes (0,00% difference)

```
Neudoerfl      SSDDNKLAYFLLTLCSLAGLVAA                                        2259
Salland        SSDDNKLAYFLLTLCSLAGLVAA                                        2259
               ***********************
```

Non-structural protein NS4b (2260-2511) – 6 amino acid changes (2,38% difference)

```
Neudoerfl      NEMGFLEKTKADLSTALWSEREEPRPWSEWTNVDIQPARSWGTYVLVVSLFTPYIIHQLQ   2319
Salland        NEMGFLEKTKADLSTVLWSEQEEPRPWSEWTNVDIQPARSWGTYVLVVSLFTPYIIHQLQ   2319
               ***************.****:***************************************

Neudoerfl      TKIQQLVNSAVASGAQAMRDLGGGAPFFGVAGHVMTLGVVSLIGATPTSLMVGVGLAALH   2379
Salland        TKIQQLVNSAVASGAQAMRDLGGGAPFFGVAGHVMTLGVVSLVGATPTSLMVGVGLAAFH   2379
               ******************************************:***************:*

Neudoerfl      LAIVVSGLEAELTQRAHKVFFSAMVRNPMVDGDVINPFGEGEAKPALYERKMSLVLATVL   2439
Salland        LAIVVSGLEAELTQRAHKVFFSAMVRNPMVDGDVINPFGEGEAKPALYERKMSLVLAIAL   2439
               ********************************************************* .*

Neudoerfl      CLMSVVMNRTVASITEASAVGLAAAGQLLRPEADTLWTMPVACGMSGVVRGSLWGFLPLG   2499
Salland        CLMSVVMNRTVASITEASAVGLAAAGQLLRPEADTLWTMPVACGMSGVVRGSLWGFLPLG   2499
               ************************************************************

Neudoerfl      HRLWLRASGGRR                                                   2511
Salland        HRLWLRASGGRR                                                   2511
               ************
```

Non-structural protein NS5 (2512-3414) – 26 amino acid changes (2,88% difference)

```
Neudoerfl      GGSEGDTLGLDWKRRLNNCTREEFFVYRRTGILETERDKARELLRRGETNVGLAVSRGTA   2571
Salland        GGSEGDTLGLDWKRRLNNCTREEFFVYRRTGILETERDRARELLRRGETNMGLAVSRGTA   2571
               **************************************:***********:*********

Neudoerfl      KLAWLEERGYATLKGEVVDLGCGRGGWSYYAASRPAVMSVRAYTIGGKGHEAPKMVTSLG   2631
Salland        KLAWLEERGYATLKGEVVDLGCGRGGWSYYAASRPAVMSVKAYTIGGRGHEAPKMVTSLG   2631
               ****************************************:******:************

Neudoerfl      WNLIKFRSGMDVFSMQPHRADTVMCDIGESSPDAAVEGERTRKVILLMEQWKNRNPTAAC   2691
Salland        WNLIKFRSGMDVFSMQPHRADTVMCDIGESNPDATVEGERTRRVILLMEQWKNRNPTAAC   2691
               ******************************.***:*******:*****************

Neudoerfl      VFKVLAPYRPEVIEALHRFQLQWGGGLVRTPFSRNSTHEMYYSTAVTGNIVNSVNVQSRK   2751
Salland        VFKVLAPYRPEVIEALHRFQLQWGGGLVRTPFSRNSTHEMYYSTAVTGNIVNSVNVQSRK   2751
               ************************************************************

Neudoerfl      LLARFGDQRGPTKVPELDLGVGTRCVVLAEDKVKEQDVQERIRALREQYSETWHMDEEHP   2811
Salland        LLARFGDQRGPTRVPELDLGVGTRCVVLAEDKVKEQDVQERIRALREQYNETWHMDEEHP   2811
               ************:************************************.**********

Neudoerfl      YRTWQYWGSYRTAPTGSAASLINGVVKLLSWPWNAREDVVRMAMTDTTAFGQQRVFKDKV   2871
Salland        YRTWQYWGSYRTAPTGSAASLINGVVKLLSWPWNAREDVVRMAMTDTTAFGQQRVFKEKV   2871
               *********************************************************:**

Neudoerfl      DTKAQEPQPGTRVIMRAVNDWILERLAQKSKPRMCSREEFIAKVKSNAALGAWSDEQNRW   2931
Salland        DTKAQEPHPGTRVIMRAVNDWILERLAQKSKPRMCSKEEFIAKVKSNAALGAWSDEQNRW   2931
               *******:****************************:***********************

Neudoerfl      ASAREAVEDPAFWRLVDEERERHLMGRCAHCVYNMMGKREKKLGEFGVAKGSRAIWYMWL   2991
Salland        ASAREAVEDPAFWHLVDEERERHLMGRCAHCVYNMMGKREKKLGEFGVAKGSRAIWYMWL   2991
               *************:**********************************************

Neudoerfl      GSRFLEFEALGFLNEDHWASRESSGAGVEGISLNYLGWHLKKLSTLNGGLFYADDTAGWD   3051
Salland        GSRFLEFEALGFLNEDHWASRESSGAGVEGISLNYLGWHLKKLSALNGGLFYADDTAGWD   3051
               ********************************************:***************

Neudoerfl      TKVTNADLEDEEQILRYMEGEHKQLATTIMQKAYHAKVVKVARPSRDGGCIMDVITRRDQ   3111
Salland        TKVTNADLEDEEQILRYMEGEHRQLAATIMQKAYHAKVVKVARPSRDGGCIMDVITRRDQ   3111
               **********************:***:*********************************

Neudoerfl      RGSGQVVTYALNTLTNIKVQLIRMMEGEGVIEAADAHNPRLLRVERWLKEHGEERLGRML   3171
Salland        RGSGQVVTYALNTLTNIKVQLIRMMEGEGVIEAADAHNPRLLRVEHWLKEHGEERLGRML   3171
               *********************************************:**************
```

```
Neudoerfl      VSGDDCVVRPLDDRFGKALYFLNDMAKTRKDIGEWEHSAGFSSWEEVPFCSHHFHELVMK   3231
Salland        VSGDDCVVRPMDDRFGKALYFLNDMAKTRKDIGEWEHSAGFSSWEEVPFCSHHFHELVMK   3231
               **********:*************************************************

Neudoerfl      DGRTLVVPCRDQDELVGRARISPGCGWSVRETACLSKAYGQMWLLSYFHRRDLRTLGLAI   3291
Salland        DGRTLVVPCRDQDELVGRARVSPGCGWSVRETACLSKAYGQMWLLSYFHRRDLRTLGLAI   3291
               ********************:***************************************

Neudoerfl      NSAVPADWVPTGRTTWSIHASGAWMTTEDMLDVWNRVWILDNPFMQNKERVMEWRDVPYL   3351
Salland        NSAVPVDWVPTGRTTWSIHASGAWMTTEDMLDVWNRVWILDNPFMHNKEKVMEWRDVPYL   3351
               *****.***************************************:***:**********

Neudoerfl      PKAQDMLCSSLVGRRERAEWAKNIWGAVEKVRKMIGPEKFKDYLSCMDRHDLHWELRLES   3411
Salland        PKAQDMVCSSLVGRKERAEWAKNIWGAVEKVRKMMGPEKFKDYLSCMDRHDLHWELKLES   3411
               ******:*******:*******************:*********************:***

Neudoerfl      SII                                                            3414
Salland        SII                                                            3414
               ***
```

Figure S1: Amino acid sequence comparison of the complete viral polyprotein of the novel TBEV-Eu strain Salland and the TBEV-Eu reference strain Neudoerfl. Polyprotein sequences of TBEV-Eu strains Salland and Neudoerfl (GenBank accession numbers BAV60898 and NP_043135, respectively) were downloaded from GenBank and aligned using Clustal Omega. Individual viral proteins were annotated based on the TBEV-Eu reference sequence. The number of substitutions are indicated for all individual viral proteins.

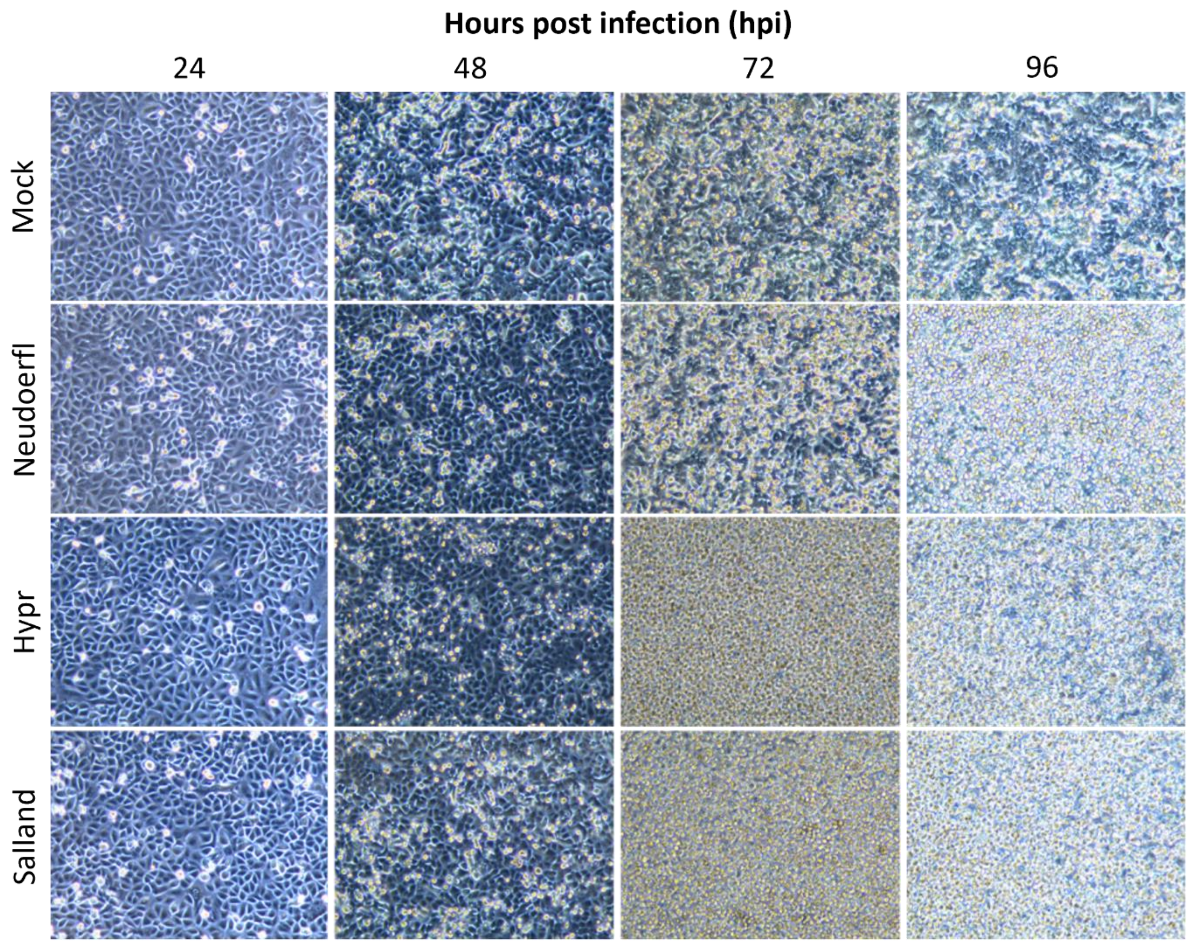

Figure S2: Representative images depicting TBEV-induced CPE on A549 cells at different time points post infection. All images were taken and processed using similar settings. CPE is visible as rounding and detachment of the cells.

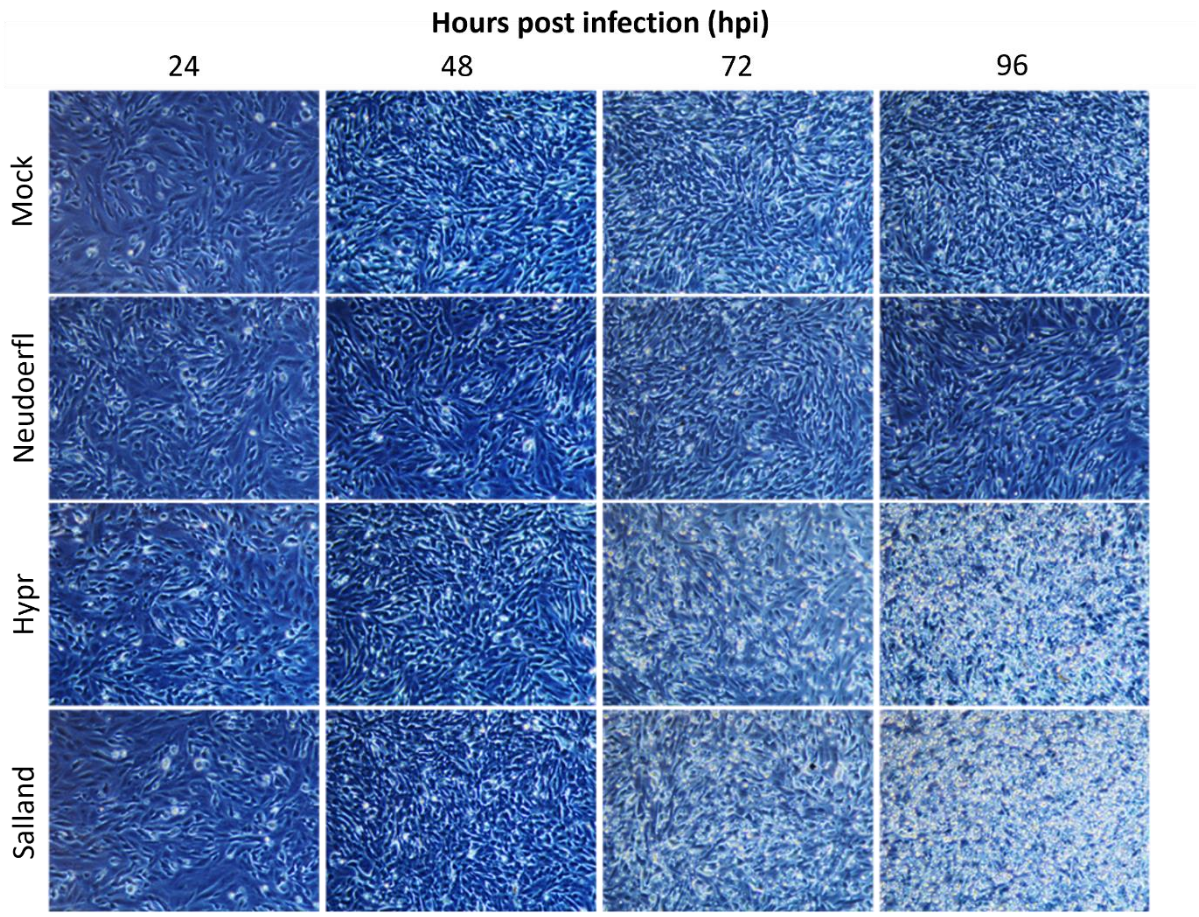

Figure S3: Representative images showing TBEV-induced CPE on SK-N-SH cells at different time points post infection. All images were taken and processed using similar settings. CPE is visible as rounding and detachment of the cells.

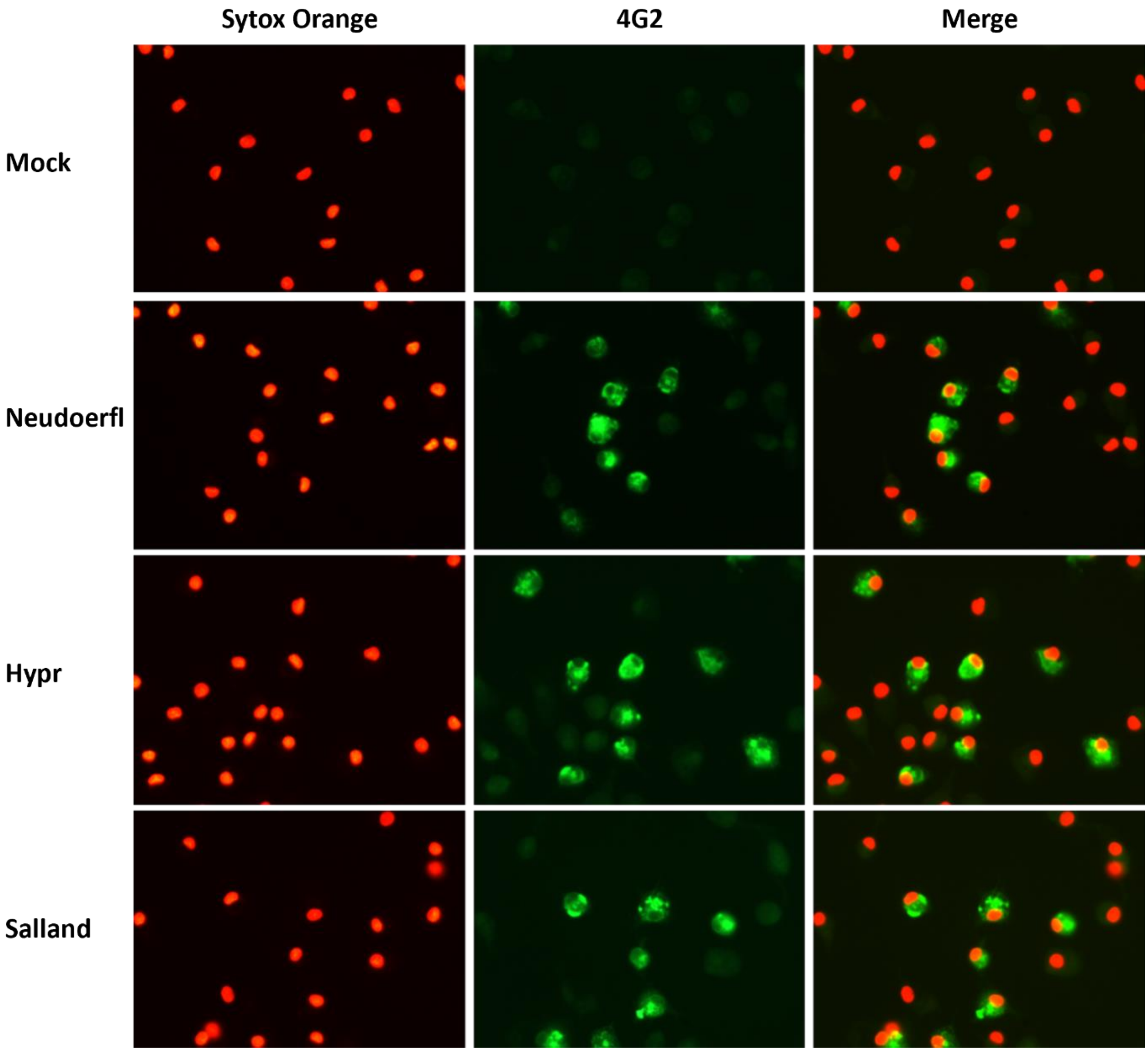

Figure S4: Representative images showing 4G2 and Sytox orange staining in moDCs. All images were captured and processed using similar settings.
